# Comparison of multiple non‐invasive methods of measuring cardiac output during pregnancy reveals marked heterogeneity in the magnitude of cardiac output change between women

**DOI:** 10.14814/phy2.13223

**Published:** 2017-04-24

**Authors:** John W. Petersen, Jing Liu, Yueh‐Yun Chi, Melissa Lingis, R. Stan Williams, Alice Rhoton‐Vlasak, Mark S. Segal, Kirk P. Conrad

**Affiliations:** ^1^ Division of Cardiovascular MedicineUniversity of FloridaGainesvilleFlorida; ^2^ Department of BiostatisticsUniversity of FloridaGainesvilleFlorida; ^3^ Division of Nephrology, Hypertension and Renal TransplantationUniversity of FloridaGainesvilleFlorida; ^4^ Department of Obstetrics and GynecologyUniversity of FloridaGainesvilleFlorida; ^5^ Nephrology and Hypertensive SectionMedical ServiceNorth Florida/South Georgia Veterans Health SystemGainesvilleFlorida; ^6^ Department of Physiology and Functional GenomicsUniversity of FloridaGainesvilleFlorida

**Keywords:** Cardiac Output, echocardiography, pregnancy

## Abstract

Various non‐invasive methods are available to measure cardiac output (CO) during pregnancy. We compared serial measures of CO using various methods to determine which provided the least variability. Ten patients with spontaneous pregnancy had estimation of CO at baseline prior to becoming pregnant and at the end of the first and third trimesters. Echocardiographic data were used to estimate CO using the Teichholz method, Simpson's biplane method, and the Doppler determined velocity time integral (VTI) method. In addition, a Bioz Dx device was used to estimate CO by impedance cardiography. CO estimated with the VTI method had the lowest beat‐to‐beat variability. CO estimated with the VTI method was higher than CO estimated with the 2D‐Teichholz method and Simpson's method. The percent change in CO during pregnancy was similar for all echo methods (VTI, Teichholz, and Simpson's biplane). Baseline CO determined with impedance cardiography was higher than CO determined with the VTI method. However, change in CO during pregnancy was significantly lower when measured with impedance cardiography. There was marked heterogeneity in the degree of rise in CO during the first trimester (−3 to 55%). The wide variation in the gestational rise in CO was unexpected, and at least in part secondary to variable increase in heart rate. We recommend the use of the Doppler determined VTI method for the estimation of CO in pregnancy.

## Introduction

The cardiovascular adaptations that occur in women during pregnancy have been extensively investigated. In particular, cardiac output (CO) rises significantly (Robson et al. 1989), but there is considerable variation in the timing and magnitude of the increase reported in the literature as determined by various non‐invasive echocardiographic methodologies (Table**** 1) (Mashini et al. 1987; Capeless and Clapp 1989; Robson et al. 1989; Duvekot et al. 1993; Mabie et al. 1994; Gilson et al. 1997; Poppas et al. 1997; Spaanderman et al. 2000). For example, the reported mean percent increase in CO during the first trimester ranges from 13% to 45% among these studies. One likely contribution to these widely varying responses is that each of the echocardiographic methods used to measure cardiac output in pregnancy—Teichholz, Simpson's, or velocity‐time integral—has inherent limitations, built in assumptions, different complexity of analysis, and variable reproducibility. Of note, in no previous investigations have all three of these echocardiographic approaches been assessed simultaneously in the same pregnant woman, and then compared. Nor has beat‐to‐beat variability for these different methods been reported in pregnancy. Finally, only mean values were previously reported, thus precluding a clear picture of the degree of heterogeneity in the gestational rise in CO among the different women within each study (Mashini et al. 1987; Capeless and Clapp 1989; Robson et al. 1989; Duvekot et al. 1993; Mabie et al. 1994; Gilson et al. 1997; Poppas et al. 1997; Spaanderman et al. 2000).

**Table 1:** Changes in cardiac output in normal human pregnancy

Ref.	No.	Method	Position	Control	Maternal age (year)	Gestational age (week)	Cardiac output (L/min) (%↑)	Heart rate (b/min) (%↑)	Stroke volume (ml/b) (%↑)
Mashini et al. 1987	16	Teichholz/5 cardiac cycles	Left Lateral	Postpartum (8 week)	Not reported	28	4.3→5.5 **(31)**	66→88 (33)	59→68 (3)
Robson et al. 1989	13	VTI/8‐10 cardiac cycles.	Semi‐Left Lateral	Pre‐pregnant	28	12	4.9→6.7 **(37)**	75→83 (11)	66→83 (26)
						32	4.9→7.3 **(49)**	75→88 (17)	66→85 (29)
Capeless and Clapp 1989	8	Teichholz/9–12 cardiac cycles	Comfortable left lateral	Pre‐pregnant	31	8	4.2→ 5.2 **(24)**	65→ 68 (5)	65→ 79 (22)
						16 24	4.2→ 5.9 **(41)** 4.2→ 5.7 **(36)**	65→ 72 (11) 65→ 73 (12)	65→ 83 (28) 65→ 81 (25)
Duvekot et al. 1993	10	VTI/5 cardiac cycles	Semi‐Left Lateral	Postpartum (12 week or after breast feeding)	29	9	4.8→ 6.1 **(28)**	66→ 78 (18)	73→ 78 (8)
						25	4.8→ 6.5 **(35)**	66→ 87 (32)	73→ 72 (−1)
Mabie et al. 1994	18	VTI/5 cardiac cycles	Left Lateral Head ↑ 15%	Postpartum (12 week)	23	12–15	5.7→ 6.9 **(21)**	69→ 79 (15)	84→ 86 (2)
						36–39	5.7→ 8.7 **(53)**	69→ 88 (28)	84→ 99 (18)
Poppas et al. 1997	16	VTI/3 representative cardiac cycles	Left Lateral	Postpartum (>6mo)	32	1st Tri	6.0→ 6.8 **(13)**	65→ 70 (8)	90→ 95 (6)
						2nd Tri 3rd Tri	6.0→ 7.6 **(27)** 6.0→ 7.9 **(32)**	65→ 77 (19) 65→ 80 (23)	90→ 99 (10) 90→ 99 (10)
Gilson et al. 1997	76	Simpson/3 cardiac cycles	Left Lateral Head ↑ 15%	Postpartum (6 week)	21	15	4.2→ 5.0 **(19)**	67→ 75 (12)	62→ 66 (7)
						26 36	4.2 → 5.7 **(36)** 4.2→ 5.8 **(38)**	67 → 83 (24) 67→ 82 (22)	62→ 69 (11) 62 → 70 (13)
Spaanderman et al. 2000	12	VTI/5 cardiac cycles	Semi‐left lateral	Pre‐pregnant	29	12	5.2→ 6.1 **(17)**	66→ 71 (8)	79→ 80 (1)

Many methods are available for the estimation of CO. Echocardiography provides several methods of measuring CO non‐invasively and is a convenient platform for measuring CO in pregnant subjects as it carries no known risk to mother or baby and is available in almost all Medical Centers. Two‐dimensional (2D) echocardiography can be used to estimate stroke volume and CO using either the Teichholz or Simpson's biplane methods. The Teichholz method provides estimation of left ventricular (LV) end‐diastolic volume (EDV) and LV end‐systolic volume (ESV) using only the internal diameter of the minor axis of the LV (Teichholz et al. 1976). Simpson's method of discs requires tracing of the endocardial border of the LV in two orthogonal views at end‐systole and end‐diastole. These measures are then used to create a series of cylinders that encompass the entire LV volume. The volume of these cylinders is summated and provides left ventricular EDV and ESV from which stroke volume and CO can be determined (Schiller et al. 1979).

Doppler echocardiography allows various methods for measuring LV stroke volume that, unlike 2D echocardiography methods, do not require assumptions of LV geometry. Pulsed wave Doppler can record the velocity‐time integral (VTI) in a defined region of interest. Previous work has shown that multiplying the VTI of the LV outflow tract, just below the aortic annulus, by the cross‐sectional area of the LV outflow tract provides estimation of stroke volume that correlates with invasive measures of stroke volume and CO in the non‐pregnant population (Lewis et al. 1984).

Impedance cardiography is another non‐invasive method of determining CO. The electrical impedance of the thorax is recorded with sensors on the neck and chest. This impedance is used to derive CO. Many studies have shown that CO determined with impedance cardiography correlates with CO determined with various invasive and non‐invasive methods at least in the non‐pregnant population (Boer et al. 1979).

The aim of this study was to obtain serial measurements of CO before and during uncomplicated pregnancies employing all three echocardiographic methods as outlined above, in order to compare the resulting CO and beat‐to‐beat variability of each approach. Our ultimate objective was to determine which method should be used in our future evaluations of CO in pregnant women conceiving by assisted reproductive technology (Conrad and Baker 2013). In addition, we also measured CO by impedance cardiography to compare this relatively rapid and easy‐to‐use methodology to echocardiography and to assess its reliability in our hands.

## Methods

### Patients

After written informed consent, 10 subjects with uncomplicated singleton pregnancies were included in this study approved by the University of Florida Institutional Review Board (IRB # 542‐2011). The subjects were first evaluated pre‐pregnancy during the follicular phase, 9.6 ± 0.5 days (mean ± SE) after the first day of the last menstrual period, and during pregnancy at 12.0 ± 0.3 and 34.2 ± 0.3 weeks of gestation. We chose these gestational ages as they are near the end of the first and third trimesters, when CO has been reported in most publications to have reached a peak and a plateau, respectively. After an overnight fast, the subjects reported to the Clinical Research Center at 0800 h. After phlebotomy and several non‐invasive vascular tests including EndoPat (which utilizes a balloon‐filled probe on the index finger to detect pulse) and SphygmoCor (which utilizes non‐invasive tonometry at the radial artery in conjunction with the brachial blood pressure to predict central blood pressure) followed by a light snack of juice and crackers, the echocardiographic study was started at 1200 h.

### Echocardiography

Echocardiograms were obtained in the left lateral decubitus position with an iE33 (Philips, The Netherlands) equipped with a broadband S5‐1 transducer (frequency transmitted 1.7 MHz, received 3.4 MHz). CO is known to be significantly lower in the supine position as compared to the left lateral position (Clark et al. 1991). Therefore, we elected to acquire echocardiographic data in the left lateral position, similar to the position done in prior studies of hemodynamics during pregnancy (Table 1). Parasternal long‐ and short axis views and three standard apical views (4‐chamber, 2‐chamber, and 3‐chamber) were obtained. M‐mode was obtained at the level of the mitral chordae. Both M‐mode and 2D parasternal long‐axis views were used to measure the LV end‐diastolic and end‐systolic dimensions used to estimate stroke volume with the Teichholz method. LV end‐systolic and end‐diastolic volumes and ejection fraction were also determined by manual tracing of the end‐systolic and end‐diastolic endocardial borders using apical 4‐ and 2‐chamber views, employing Simpson's biplane method. Doppler was performed from an apical 5‐chamber orientation. Pulsed‐wave Doppler with placement of sample volume in the left ventricular outflow tract immediately proximal to the aortic valve cusps was used to determine LV outflow tract VTI. LV outflow VTI was measured in up to five different beats. All echo measures were not performed on sequential beats but instead obtained over a period of at least one minute, in order to mitigate the respiratory variation in subjects who were breathing normally at rest. LV outflow tract cross‐sectional area was estimated after measuring the diameter of the outflow tract on parasternal images. LV outflow cross‐sectional area was measured in up to five different beats. An experienced echocardiographer (JP) identified the single best VTI (typically the largest VTI with appropriate Doppler profile) and the single best LV ouflow diameter (typically the largest well visualized diameter). VTI data was used to estimate stroke volume and CO in different ways. (1) The VTI of each beat was multiplied by the estimated average LV outflow cross‐sectional area (determined by averaging the measured radiuses of all beats). The average CO for up to five beats was then determined (aD). (2) The VTI of each beat was multiplied by the best LV outflow cross‐sectional area determined from the single best LV outflow diameter. The average CO for up to five beats was then determined (bD). (3) The single best VTI was multiplied by the average LV outflow cross sectional area of all beats (bVaD). (4) The single best VTI was multiplied by the single best LV outflow cross‐sectional area (bVbD).

### Impedance cardiography

Impedance Cardiography (ICG) measurements were performed using the BioZ Dx device (SonoSite CardioDynamics, San Diego, CA) with subjects lying in supine position after at least 5 minutes of rest. Briefly, a pair of sensors placed on either side of the neck and a second pair of sensors placed on either side on the mid‐axillary line at the level of the xiphoid process are used to transmit a high frequency, low amplitude alternating current. The BioZ Dx calculates stroke volume based on detected change in impedance across the thoracic cavity over multiple cardiac cycles. This calculation includes adjustments to correct for differences in thoracic volume derived from sex, height and body weight, as well as changes in volume caused by respiration.

### Statistics

Data are presented as mean ± SE (standard error) unless otherwise indicated. The non‐parametric Wilcoxon sign‐rank tests were performed to assess statistical differences between paired measurements. Pearson correlations (*r*) and coefficient of determination (*R* ^*2*^) were computed to quantify the extent of correlation and percentage of variance explained. For simple linear regressions, *r* ^*2*^ = *R* ^*2*^, which can be tested using the *t* distribution. Semi‐partial correlation was also computed to account for the effects of confounding factors. Of note, in order to evaluate beat‐to‐beat variability of VTI methods, we examined the standard deviation of estimated stroke volume determined by multiplying each VTI by the average estimated LV outflow cross‐sectional area (aD) and by multiplying each VTI by the cross‐sectional area determined from the single best LV outflow diameter (bD).

## Results

### Subjects

Baseline demographics and clinical characteristics of the 10 subjects with singleton pregnancies are shown in Table**** 2 **.** Although the subjects were chosen randomly from a larger population of women studied, all 10 were white and non‐Hispanic. They were healthy and experienced uncomplicated pregnancies. Inspection of prescribed medications or other substances did not reveal any known to significantly impact cardiac function or systemic hemodynamics. All subjects were taking a prenatal vitamin. One subject was taking Flonase.

**Table 2:** Subject demographic and clinical characteristics

** **	Count	Mean ± SE	Range
Age (year) at baseline	10	32.5 ± 2.01	24.08–44.5
Age (year) at pregnancy	10	32.8 ± 1.96	24.82–44.58
Weight (kg)	10	68 ± 6.5	44.9–115.4
Weight (lbs)	10	150 ± 14.3	98.99–254.4
BMI	10	25 ± 2.36	17.6–43.1
Systolic blood pressure at delivery (mmHg)	10	128 ± 2	117–139
Diastolic blood pressure at delivery (mmHg)	10	71.7 ± 3	57.7–86.5
Birth Weight (g)	10	3419 ± 163	2574–4145
Race/ethnicity			
White, non‐Hispanic or non‐Latino	10		
Smoking status			
Yes	1 (only at baseline)		
No	9		
Gravida/parity			
G0P0	6		
G1P1	2		
G2P0	1		
G3P1	1		
Baby sex			
Female	6		
Male	4		
Apgar score (1′, 5′; scale 1–9)			
9,9	4		
8,9	5		
1,9	1		

### Comparison of methods for determining cardiac output

#### VTI methods

**Figure 1:**
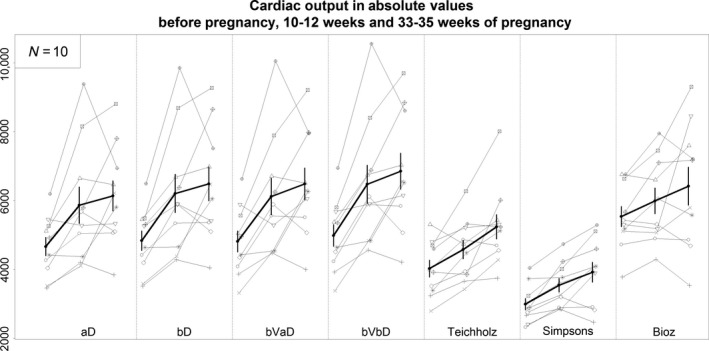
Cardiac output (mL/min) for each subject (thin lines) pre‐pregnancy, 10–12 weeks of pregnancy, and 33–35 weeks of pregnancy. Thick line is average (±SE) cardiac output for all subjects. Abbreviations are same as Table 3.

**Figure 2:**
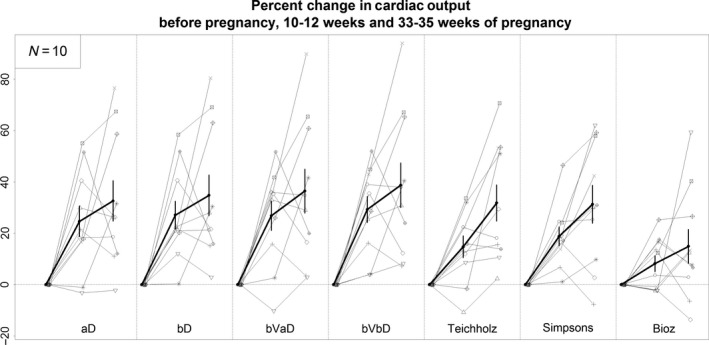
Percent change cardiac output for each subject (thin lines) from pre‐pregnancy to 10–12 weeks of pregnancy and 33–35 weeks of pregnancy. Thick line is average (±SE) percent change in cardiac output for all subjects. Abbreviations are same as Table 3.

There was no significant difference in absolute estimated cardiac output among the various VTI methods used (Table**** 3 **,** Fig.**** 1). For example, the estimated CO determined by averaging the product of each VTI by the average LV outflow cross‐sectional area (aD) was not significantly different than the estimated CO determined by multiplying the best VTI by the best LV outflow cross‐sectional area (bVbD) before pregnancy (4.7 vs. 5.0 L/min). Similarly, the percent rise in CO during pregnancy and linear change over time was not different among VTI methods (Table**** 4 **,** Fig.**** 2). At the end of the first trimester estimated CO determined with aD and bVbD VTI methods increased 24.7% and 29.3%, respectively. Similarly, by the end of the third trimester estimated CO as determined by these two approaches increased 32.6% and 38.7%, respectively.

**Table 3:** Estimated cardiac output (L/min) using various methods

Method	Before pregnancy				10–12 weeks of pregnancy				33–35 weeks of pregnancy			
	Mean	SE	Min	Max	Mean	SE	Min	Max	Mean	SE	Min	Max
VTI‐aD	4.7	0.26	3.5	6.2	5.9	0.52	4.1	9.4	6.1	0.44	3.8	8.8
VTI‐bD	4.8	0.28	3.5	6.5	6.2	0.55	4.3	9.9	6.5	0.49	4.0	9.3
VTI‐bVaD	4.8	0.30	3.3	6.6	6.1	0.53	4.5	10.1	6.5	0.46	4.0	9.2
VTI‐bVbD	5.0	0.31	3.4	6.9	6.5	0.55	4.6	10.6	6.9	0.52	4.2	9.7
Teichholz	4.0	0.24	2.8	5.3	4.6	0.27	3.4	6.3	5.2	0.35	3.7	8.0
2D Teich.	3.7	0.27	2.4	5.1	4.2	0.30	2.9	6.1	4.7	0.39	3.4	7.9
M Teich.	4.4	0.21	3.4	5.7	5.0	0.29	3.7	6.5	5.8	0.35	3.8	8.1
Simpson's	3.0	0.16	2.3	4.0	3.5	0.19	2.9	4.7	3.9	0.29	2.5	5.3
ICG (BioZ)	5.5	0.29	3.8	6.8	6.0	0.37	4.3	8.0	6.4	0.55	3.5	9.3

VTI, Velocity Time Integral; aD, average VTI x average LV outflow diameter; bD, average VTI × single best LV outflow diameter; bVaD, single best VTI × average LV outflow diameter; bVbD, single best VTI x single best LV outflow diameter; 2D Teich, 2D images used for end‐systolic and end‐diastolic measures used in Teichholz equation; M Teich, M mode images used for end‐systolic and end‐diastolic measures used in Teichholz equation; Teichholz, average of 2d and M mode measures; ICG, Impedance Cardiography.

**Table 4:** Percent change (%) in cardiac output from baseline (before pregnancy)

	% Change from baseline to week 10–12 of pregnancy				% Change from baseline to week 33–35 of pregnancy			
Method	Mean	SE	Min	Max	Mean	SE	Min	Max
VTI‐aD	24.7	5.9	−3.2	55	32.6	7.9	−2.2	76.6
VTI‐bD	27.1	5.4	0.3	58.4	34.8	7.9	2.9	80.4
VTI‐bVaD	26.8	5.7	−10.2	51.7	36.4	8.5	2.9	89.9
VTI‐bVbD	29.3	5	3.9	52	38.7	8.6	7.3	94
Teichholz	14.7	4.2	−10.8	33.6	31.8	7	2.3	70.7
2D Teich.	14.2	5.6	−23.2	36.7	29.6	8.1	−14.5	68.8
M Teich.	13.7	4.5	−4.9	39.4	31.9	7.8	2.7	71.9
Simpson's	18.8	3.6	1.1	46.5	31.3	7.4	−7.7	62.1
ICG (BioZ)	8.1	3	−2.4	25.3	14.9	6.6	−13.7	59.4

VTI, Velocity Time Integral; aD, average VTI x average LV outflow diameter; bD, average VTI × single best LV outflow diameter; bVaD, single best VTI × average LV outflow diameter; bVbD, single best VTI × single best LV outflow diameter; 2D Teich, 2D images used for end‐systolic and end‐diastolic measures used in Teichholz equation; M Teich, M mode images used for end‐systolic and end‐diastolic measures used in Teichholz equation; Teichholz, average of 2d and M mode measures, ICG, Impedance Cardiography.

#### Teichholz methods

End‐systolic and end‐diastolic LV dimensions were determined using both 2D and M‐mode images and entered into the Teichholz equation to estimate stroke volume and CO. CO was higher before pregnancy when using M‐mode measures as compared to 2D images (4.4 vs. 3.7 L/min, *P* < 0.01), and this difference between the two approaches persisted during pregnancy (Table**** 3 **,** Fig.**** 1). CO estimated with M‐mode Teichholz technique was not significantly different than CO estimated with the Doppler method (VTI‐aD); however, baseline CO estimated with 2D Teichholz technique was lower than baseline CO estimated with Doppler‐VTI‐aD (3.7 vs. 4.7 L/min, *P* = 0.002). Nevertheless, the change in CO during pregnancy and linear change over time was not significantly different between M‐mode and 2D Teichholz methods (Table**** 4 **,** Fig.**** 2).

#### Simpson's method

The estimated CO was also different between the VTI and Simpson's method (Table**** 3 **,** Fig.**** 1). CO measured by VTI was higher than CO estimated with Simpson's biplane method. Most notably, CO estimated with Simpson's was approximately 35% lower than CO estimated with VTI methods at every time point. However, the percentage change during pregnancy and linear change over time was not significantly different between the different echo methods (Table**** 4 **,** Fig.**** 2).

#### Impedance cardiography

CO estimated at baseline in the pre‐pregnant condition with impedance cardiography was greater than CO estimated with VTI methods (*P* = 0.004; Table**** 3 **,** Fig.**** 1). The percent change in CO during pregnancy (Table**** 4 **,** Fig.**** 2) was higher for VTI‐aD than BioZ in both the first trimester (BioZ: 8.1 ± 3.0 vs VTI‐aD: 24.7 ± 5.9% and at the end of the third trimester (BioZ: 14.9 ± 6.6 vs. VTI‐aD: 32.6 ± 7.9%), although statistical significance was borderline (both differences *P* = 0.064).

### Beat‐to‐beat variability in estimated cardiac output

Despite sinus rhythm in all patients, we found considerable beat‐to‐beat variability in estimated stroke volume. However, beat‐to‐beat variability was ~2‐times greater for Teichholz and Simpson's biplane compared to VTI method before and during pregnancy (Table**** 5).

**Table 5:** Beat‐to‐beat variability, measured by the mean of standard deviations (SD) across beats for the same patient

	Before pregnancy		10–12 weeks of pregnancy		33–35 weeks of pregnancy		All visits
	beats	Mean SD (mL)	beats	Mean SD (mL)	beats	Mean SD (mL)	Mean SD (mL)
aD CO	3–5^a^	282	5	335	5	454	364
bD CO	3–5^a^	291	5	352	5	480	382
Teichholz CO	5	528	5	713	5	844	707
Simpson's CO	5	437	5	388	5	835	589

a 3 beats from one patient, 4 beats from three patients, and 5 beats from six patients.

aD CO, mean standard deviation of CO for each patient determined by multiplying VTI of each beat by average LV outflow area; bD CO, mean standard deviation of CO for each patient determined by multiplying VTI of each beat by single best LV outflow area.

### Mechanism of increased cardiac output

**Figure 3:**
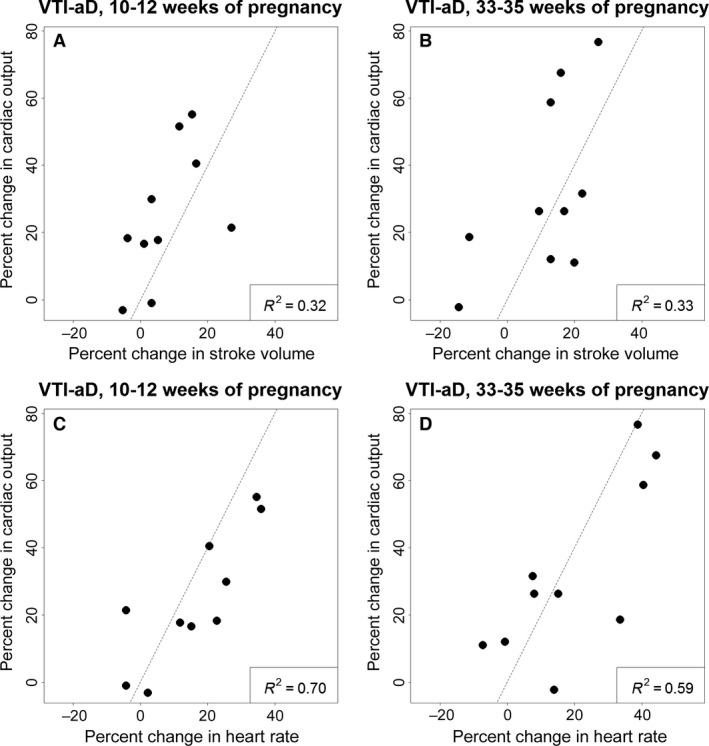
Correlation between percent change in cardiac output and stroke volume and heart rate from pre‐pregnancy to 10–12 weeks of pregnancy (A, C) and to 33–35 weeks pregnancy (B, D). Percent change in heart rate (C, D) has a stronger correlation with percent change in cardiac output than does stroke volume at both gestational stages (A, B).

The gestational rise in CO is a consequence of increase in heart rate, stroke volume or both. Further, stoke volume and heart rates are likely significantly impacted by systemic vascular resistance (SVR). Mean arterial pressure, heart rate, stroke volume (determined with VTI‐aD method), and SVR for our subjects at the various visits are shown in Table**** 6. While the average CO for all subjects increased in a way that was similar to many previously reported investigations (Table**** 1), there was an impressive degree of variability in the change in CO during pregnancy among individuals (Fig.**** 2). In our cohort, increase in heart rate had stronger correlation with changes in cardiac output than stroke volume (Fig.**** 3). Further, a semi‐partial correlation demonstrated that change in heart rate had the strongest correlation with change in CO during the first trimester of pregnancy, even when controlling for changes in SV and SVR (*r* = 0.71).

**Table 6:** Mean arterial pressure, heart rate, stroke volume and systemic vascular resistance

	Before pregnancy		10–12 weeks of pregnancy		33–35 weeks of pregnancy	
	Mean	SE	Mean	SE	Mean	SE
MAP (mmHg)	72.7	1.8	69	1.7	72.3	1.5
HR (bpm)	64.8	1.7	75	3.1	76.9	3.0
SV (VTI‐aD method, mL)	72.4	4.3	77.7	5.0	80.3	5.3
SVR (mmHg min/L)	1283.9	72.3	996.2	65.8	985.6	65.3

## Discussion

Accurate measures of CO are important for the ongoing study of cardiovascular adaptations to pregnancy. We found that CO estimated with VTI methods had the least beat‐to‐beat variability, and the percentage change in CO during pregnancy was not different between the various echo methods. However, absolute CO estimated with VTI methods was larger than CO estimated with most other echocardiographic methods. The absolute CO estimated with the VTI method in our cohort is similar to the absolute CO measured in other studies evaluating pregnant subjects (Table 1). Also, absolute CO in our study was similar to that determined in one of the few studies that compared CO determined by VTI method with CO measured with invasive techniques in pregnant patients (Lee et al. 1988).

Doppler determined VTI methods are known to have good inter‐observer reliability (Lewis et al. 1984). This likely is related to the ease of measuring LV outflow tract diameters and LV outflow pulse‐wave Doppler profiles, which contribute to lower beat to beat variability. The Teichholz method has shown to correlate with many invasive and non‐invasive methods of determining LV chamber volumes and stroke volume (Folland et al. 1979; Arora et al. 2010), but this method is dependent on assumptions regarding the shape of the LV and the relation of the minor axis diameter of the LV to the length of the LV. In subjects with relatively uniform contractility throughout the LV, the Teichholz method can provide a reasonable estimate of CO. We demonstrated that estimated CO determined by the Teichholz method using the M‐Mode measures of LV dimensions was similar to CO determined with VTI methods (Table**** 3 **,** Fig.**** 1). LV end‐diastolic and end‐systolic diameters measured on 2D images led to lower estimated CO as compared to M‐mode or Doppler methods. Equations used in the Teichholz method were developed using M‐mode data. M‐mode measures are often not perpendicular to the long axis of the LV and this likely leads to larger diameters, particularly at end‐diastole, and larger estimated CO. Therefore, we recommend use of M‐mode measures when using the Teichholz method to estimate CO. The M‐mode Teichholz method may provide a reasonable measure of CO, if Doppler techniques are unavailable. However, given the improved beat‐to‐beat variability of the VTI method, we prefer the use of VTI for estimating CO (Table**** 5).

Similar to the Teichholz method, Simpson's biplane method requires geometric assumptions that can impair its estimation of CO (Folland et al. 1979; Starling et al. 1981). Comparable to other reports, we found that CO estimated with Simpson's method was systematically lower than CO estimated with the VTI method (Table**** 3 **,** Fig.**** 1). Other investigators have shown that this may relate to an underestimation of LV end‐diastolic volume with the Simpson's method (Schiller et al. 1979). Further, the apical images required for the Simpson's method were often of sub‐optimal quality in our subjects, because of their distended abdomen during pregnancy. Because of the challenge of visualizing the endocardium in many subjects, it is not surprising that this method produced more beat‐to‐beat variability.

VTI estimated CO at rest has also been shown to have limited variability day to day, and therefore is believed to provide an appropriate measure of CO over time (Moulinier et al. 1991). While beat‐to‐beat variability was low with VTI methods both before and during pregnancy (Table 5), we recommend the use of at least three VTI and LV diameter measurements for the determination of an average stroke volume, in order to maximize precision and accuracy. The averaging of stroke volume from multiple beats over a period of a minute or two can also help account for respiratory variation in stroke volume. Invasive techniques have demonstrated a 7% change in stroke volume with respiration (Ruskin et al. 1973). We did not measure sequential beats but averaged echo measures that were recorded typically over at least one minute. We felt that averaged measures at different phases of respiration provided a better representation of daily physiology, as a deep breath hold is known to impact cardiac output (Sakuma et al. 2001).

Baseline, pre‐pregnant CO was significantly greater when measured by BioZ relative to VTI methods (Table**** 3 **,** Fig.**** 1). This finding of an overestimation of SV and CO by impedance cardiography is consistent with the comprehensive review of the literature by Staelens and colleagues (Staelens et al. 2013). Possibly related to the higher baseline CO, impedance cardiography demonstrated only a small increase in CO in the first trimester and almost no additional increase in CO by the end of the third trimester (Table**** 3 **,** Fig.**** 2). Many, but not all studies have demonstrated reasonable agreement between SV and CO determined with impedance cardiography and other methods of measuring CO at single time points in pregnancy (Staelens et al. 2013). However, studies evaluating changes in impedance cardiography estimates of CO over the course of pregnancy have demonstrated an unexpected drop in estimated CO from the first to third trimester (Davies et al. 1986; Moertl et al. 2012). Others (Moertl et al. 2012; McIntyre et al. 2015) have suggested that the discrepancy in CO measured by impedance cardiography during pregnancy may relate to gestational changes in the thoracic dimensions and composition that predictably challenge standard equations used by impedance cardiography to estimate CO.

In our study, heart rate appeared to have a bigger impact on change in estimated CO during pregnancy than did change in stroke volume. Prior study using serial echo measures in pregnancy have shown that increased heart rate, especially in the later stages of pregnancy, does have a significant impact on change in CO during pregnancy (Hunter and Robson 1992). Heart rate was reliably measured with all of the methods investigated during this study. Change in heart rate may be an easy way to monitor changes in cardiovascular function during pregnancy; however, not all reports demonstrated that heart rate was the primary driver of the gestational increase in CO. Rather, SV was the major component (Capeless and Clapp 1989; Robson et al. 1989) (Table**** 1). For accurate and precise measures of CO a reliable estimate of stroke volume is required. VTI appears to offer a precise measure of stroke volume that correlates with invasive measures (Lewis et al. 1984).

Upon inspection of CO before and during pregnancy in our individual subjects, the large variability of the gestational rise in CO among the 10 subjects was striking (Figs.**** 1, 2) and unexpected or at least underappreciated, because previous studies only reported mean values. In general, heart rate and SV accounted for 70% and 30% of this variability, respectively (Fig.**** 3). We feel that both changes in preload and afterload should be considered when trying to account for the changes in heart rate, SV, and associated CO that occur during pregnancy. In our study, changes in preload did not clearly correlate with changes in CO, as there were no significant correlations between the magnitude of gestational change in CO and hematocrit, left atrial or left ventricular end diastolic dimensions—surrogates of cardiac preload and plasma volume expansion during pregnancy. In contrast, there was a significant correlation between the degree of gestational percent change in CO and absolute change in systemic vascular resistance (preconception‐first trimester: *r* ^*2*^ (squared correlation) = 0.47, *P* = 0.030; preconception‐ third trimester: *r* ^*2*^ = 0.54, *P* = 0.016). This relationship might be anticipated, because SVR is derived from CO and MAP; nevertheless, it suggests that there may be a causal relationship between CO and systemic vasodilation or cardiac afterload, another major determinant of CO. Interestingly, there were also significant correlations between the changes in CO and SVR with BMI, but only from preconception to the first trimester. Insofar as accrual of maternal body tissue including fat rather than volume expansion or fetoplacental growth accounts for much of the weight gain during the first trimester (Hytten and Chamberlain 1980), these correlations may reflect the angiogenesis‐vasculogenesis necessarily associated with tissue expansion, which could conceivably contribute to the gestational decline in SVR during the first trimester, thus abetting an increase in CO.

Our study has major advantages and limitations. The echocardiograms analyzed for this study were all acquired with the same echocardiogram system by the same experienced echo technologist. All measurements were performed by an experienced echocardiologist. The major limitation of our study was that for obvious ethical considerations no invasively determined measure of CO was available for comparison. However, each of the ultrasound methods evaluated in this study have previously been shown to correlate with invasive measures of CO (Teichholz et al. 1976; Schiller et al. 1979; Lewis et al. 1984; Staelens et al. 2013). Additionally, VTI methods have been shown to correlate with invasive measures of CO in pathologic pregnant patients (Lee et al. 1988). While our study was small, statistically significant differences were identified and are able to contribute to power calculations for future trials. The number of subjects in our study is similar to many of the other investigations evaluating the change in CO during pregnancy (Table 1).

In conclusion, stroke volume estimated with the Doppler determined VTI method provided the measure of CO with the least beat‐to‐beat variability. Doppler determined measures of CO were typically higher than CO determined with 2D‐Teichholz or Simpson's biplane method, but relative change in CO during pregnancy was similar with all echocardiographic techniques. We recommend the use of Doppler determined VTI method, but not impedance cardiography for the estimation of CO in pregnancy.

## Conflict of Interest

None declared.

## References

[phy213223-bib-0001] Arora, G. , A. M. Morss , et al. 2010 Difference in left ventricular ejection fraction using teichholz formula and volumetric methods by cmr: implications for patient stratification and selection of therapy. J. Cardiovasc. Magn. Reson. 12(Suppl1):P202.

[phy213223-bib-0002] Boer, P. , J. C. Roos , et al. 1979 Measurement of cardiac output by impedance cardiography under various conditions. Am. J. Physiol. 237:H491–H496. 495735 10.1152/ajpheart.1979.237.4.H491

[phy213223-bib-0003] Capeless, E. L. , and J. F. Clapp . 1989 Cardiovascular changes in early phase of pregnancy. Am. J. Obstet. Gynecol. 161(6 Pt 1):1449–1453. 2603897 10.1016/0002-9378(89)90902-2

[phy213223-bib-0004] Clark, S. L. , D. B. Cotton , et al. 1991 Position change and central hemodynamic profile during normal third‐trimester pregnancy and post partum. Am. J. Obstet. Gynecol. 164:883–887. 2003555 10.1016/s0002-9378(11)90534-1

[phy213223-bib-0005] Conrad, K. P. , and V. L. Baker . 2013 Corpus luteal contribution to maternal pregnancy physiology and outcomes in assisted reproductive technologies. Am. J. Physiol. Regul. Integr. Comp. Physiol. 304:R69–R72. 23100030 10.1152/ajpregu.00239.2012 PMC3543656

[phy213223-bib-0006] Davies, P. , R. I. Francis , et al. 1986 Analysis of impedance cardiography longitudinally applied in pregnancy. Br. J. Obstet. Gynaecol. 93:717–720. 3730342

[phy213223-bib-0007] Duvekot, J. J. , E. C. Cheriex , et al. 1993 Early pregnancy changes in hemodynamics and volume homeostasis are consecutive adjustments triggered by a primary fall in systemic vascular tone. Am. J. Obstet. Gynecol. 169:1382–1392. 8267033 10.1016/0002-9378(93)90405-8

[phy213223-bib-0008] Folland, E. D. , A. F. Parisi , et al. 1979 Assessment of left ventricular ejection fraction and volumes by real‐time, two‐dimensional echocardiography. A comparison of cineangiographic and radionuclide techniques. Circulation 60:760–766. 476879 10.1161/01.cir.60.4.760

[phy213223-bib-0009] Gilson, G. J. , S. Samaan , et al. 1997 Changes in hemodynamics, ventricular remodeling, and ventricular contractility during normal pregnancy: a longitudinal study. Obstet. Gynecol. 89:957–962. 9170474 10.1016/s0029-7844(97)85765-1

[phy213223-bib-0010] Hunter, S. , and S. C. Robson . 1992 Adaptation of the maternal heart in pregnancy. Br. Heart. J. 68:540–543. 1467047 10.1136/hrt.68.12.540 PMC1025680

[phy213223-bib-0011] Hytten, F. , and G. Chamberlain . 1980 Clinical physiology in obstetrics. Blackwell Scientific Publications, Oxford.

[phy213223-bib-0012] Lee, W. , R. Rokey , et al. 1988 Noninvasive maternal stroke volume and cardiac output determinations by pulsed Doppler echocardiography. Am. J. Obstet. Gynecol. 158(3 Pt 1):505–510. 3348311 10.1016/0002-9378(88)90014-2

[phy213223-bib-0013] Lewis, J. F. , L. C. Kuo , et al. 1984 Pulsed Doppler echocardiographic determination of stroke volume and cardiac output: clinical validation of two new methods using the apical window. Circulation 70:425–431. 6744546 10.1161/01.cir.70.3.425

[phy213223-bib-0014] Mabie, W. C. , T. G. DiSessa , et al. 1994 A longitudinal study of cardiac output in normal human pregnancy. Am. J. Obstet. Gynecol. 170:849–856. 8141215 10.1016/s0002-9378(94)70297-7

[phy213223-bib-0015] Mashini, I. S. , S. J. Albazzaz , et al. 1987 Serial noninvasive evaluation of cardiovascular hemodynamics during pregnancy. Am. J. Obstet. Gynecol. 156:1208–1213. 3578440 10.1016/0002-9378(87)90146-3

[phy213223-bib-0016] McIntyre, J. P. , K. M. Ellyett , et al. 2015 Validation of thoracic impedance cardiography by echocardiography in healthy late pregnancy. BMC Pregnancy Childbirth 15:70. 25886289 10.1186/s12884-015-0504-5 PMC4389339

[phy213223-bib-0017] Moertl, M. G. , D. Schlembach , et al. 2012 Hemodynamic evaluation in pregnancy: limitations of impedance cardiography. Physiol. Meas. 33:1015–1026. 22562970 10.1088/0967-3334/33/6/1015

[phy213223-bib-0018] Moulinier, L. , T. Venet , et al. 1991 Measurement of aortic blood flow by Doppler echocardiography: day to day variability in normal subjects and applicability in clinical research. J. Am. Coll. Cardiol. 17:1326–1333. 2016450 10.1016/s0735-1097(10)80143-3

[phy213223-bib-0019] Poppas, A. , S. G. Shroff , et al. 1997 Serial assessment of the cardiovascular system in normal pregnancy. Role of arterial compliance and pulsatile arterial load. Circulation 95:2407–2415. 9170404 10.1161/01.cir.95.10.2407

[phy213223-bib-0020] Robson, S. C. , S. Hunter , et al. 1989 Serial study of factors influencing changes in cardiac output during human pregnancy. Am. J. Physiol. 256(4 Pt 2):H1060–H1065. 2705548 10.1152/ajpheart.1989.256.4.H1060

[phy213223-bib-0021] Ruskin, J. , R. J. Bache , et al. 1973 Pressure‐flow studies in man: effect of respiration on left ventricular stroke volume. Circulation 48:79–85. 4781252 10.1161/01.cir.48.1.79

[phy213223-bib-0022] Sakuma, H. , N. Kawada , et al. 2001 Effect of breath holding on blood flow measurement using fast velocity encoded cine MRI. Magn. Reson. Med. 45:346–348. 11180443 10.1002/1522-2594(200102)45:2<346::aid-mrm1044>3.0.co;2-i

[phy213223-bib-0023] Schiller, N. B. , H. Acquatella , et al. 1979 Left ventricular volume from paired biplane two‐dimensional echocardiography. Circulation 60:547–555. 455617 10.1161/01.cir.60.3.547

[phy213223-bib-0024] Spaanderman, M. E. , M. Meertens , et al. 2000 Cardiac output increases independently of basal metabolic rate in early human pregnancy. Am. J. Physiol. Heart Circ. Physiol. 278:H1585–H1588. 10775137 10.1152/ajpheart.2000.278.5.H1585

[phy213223-bib-0025] Staelens, A. , K. Tomsin , et al. 2013 Non‐invasive assessment of gestational hemodynamics: benefits and limitations of impedance cardiography versus other techniques. Expert Rev. Med. Devices 10:765–779. 24195460 10.1586/17434440.2013.853466

[phy213223-bib-0026] Starling, M. R. , M. H. Crawford , et al. 1981 Comparative accuracy of apical biplane cross‐sectional echocardiography and gated equilibrium radionuclide angiography for estimating left ventricular size and performance. Circulation 63:1075–1084. 7471367 10.1161/01.cir.63.5.1075

[phy213223-bib-0027] Teichholz, L. E. , T. Kreulen , et al. 1976 Problems in echocardiographic volume determinations: echocardiographic‐angiographic correlations in the presence of absence of asynergy. Am. J. Cardiol. 37:7–11. 1244736 10.1016/0002-9149(76)90491-4

